# Metabolic syndrome score as an indicator in a predictive nomogram for lymph node metastasis in endometrial cancer

**DOI:** 10.1186/s12885-023-11053-4

**Published:** 2023-07-04

**Authors:** Xuan Feng, Xing Chen Li, Xiao Yang, Yuan Cheng, Yang Yang Dong, Jing Yuan Wang, Jing Yi Zhou, Jian Liu Wang

**Affiliations:** Department of Obstetrics and Gynecology, Peking University People’s Hospital, Beijing, 100044 China

**Keywords:** Endometrial cancer, Metabolic, Lymph node metastasis, Nomogram

## Abstract

**Background:**

Lymph node metastasis (LNM) is an important factor affecting endometrial cancer (EC) prognosis. Current controversy exists as to how to accurately assess the risk of lymphatic metastasis. Metabolic syndrome has been considered a risk factor for endometrial cancer, yet its effect on LNM remains elusive. We developed a nomogram integrating metabolic syndrome indicators with other crucial variables to predict lymph node metastasis in endometrial cancer.

**Methods:**

This study is based on patients diagnosed with EC in Peking University People’s Hospital between January 2004 and December 2020. A total of 1076 patients diagnosed with EC and who underwent staging surgery were divided into training and validation cohorts according to the ratio of 2:1. Univariate and multivariate logistic regression analyses were used to determine the significant predictive factors.

**Results:**

The prediction nomogram included MSR, positive peritoneal cytology, lymph vascular space invasion, endometrioid histological type, tumor size >  = 2 cm, myometrial invasion >  = 50%, cervical stromal invasion, and tumor grade. In the training group, the area under the curve (AUC) of the nomogram and Mayo criteria were 0.85 (95% CI: 0.81–0.90) and 0.77 (95% CI: 0.77–0.83), respectively (*P* < 0.01). In the validation group (*N* = 359), the AUC was 0.87 (95% CI: 0.82–0.93) and 0.80 (95% CI: 0.74–0.87) for the nomogram and the Mayo criteria, respectively (*P* = 0.01). Calibration plots revealed the satisfactory performance of the nomogram. Decision curve analysis showed a positive net benefit of this nomogram, which indicated clinical value.

**Conclusion:**

This model may promote risk stratification and individualized treatment, thus improving the prognosis.

**Supplementary Information:**

The online version contains supplementary material available at 10.1186/s12885-023-11053-4.

## Introduction

Endometrial cancer (EC) is one of the three malignant tumors with the highest incidence in the female reproductive system, and its incidence is increasing recently [1]. Lymphatic routes are the main metastasis mechanism of EC. The pelvic and abdominal lymph node metastasis (LNM) status is an important indicator to evaluate the prognosis of EC and guide the adjuvant therapy [2]. Patients with lymph node involvement have a higher risk of recurrence and worse overall survival compared to those without LNM [3, 4]. It has been controversial whether routine pelvic and abdominal lymph node dissection is necessary for patients with EC [5]. Some believe that comprehensive staging surgery and routine lymphadenectomy are indispensable for patients to ensure lesion excision and precise staging [6]. Nonetheless, lymph node resection may increase the risk of vascular injury, cause postoperative lymphedema of lower limbs, and other complications, affecting life quality [7]. The Mayo risk stratification model was previously used in lymphadenectomy decision-making, it defines low risk as grade I/II, endometrioid type, tumor diameter ≤ 2 cm, and myometrium invasion (MI) ≤ 50% [5]. However, Mayo criteria is limited in current clinical practice [8]. Studies reported that the overall risk of metastasis is not high and mainly occurs in cases with high-risk factors such as deep myometrium infiltration and tumor size > 2 cm [9, 10]. Gradually, comprehensive staging surgery was omitted in the low-risk group since the low-risk group demonstrated 99% 5-year survival without lymphadenectomy [11]. By 2018, National Comprehensive Cancer Network (NCCN) guidelines no longer recommend routine lymphadenectomy for clinical stage I endometrial carcinoma [12]. Currently, the sentinel lymph node biopsy (SLNB) helps to identify the first affected lymph node of cancer and conduct the histological test to determine if it is related to cancer cells, which benefits EC precise evaluation [13]. The FIRES trial reported that the sensitivity of sentinel lymph node mapping was 97.2% and the negative predictive value was 99.6%, indicating that SLNB has high diagnostic accuracy and can safely replace systematic lymph node dissection in staging surgery for endometrial cancer [14]. To date, there is no non-invasive alternative in LNM evaluation. To avoid overtreatment or misdiagnosis, accurate prediction of LNM is needed to guide the management of EC. Some researches explored predictive models. Cox Bauer et al. proposed a prediction model based on tumor diameter (≤ 50 and > 50 mm) and modified forms of MI (≤ 33%, 33–66%, > 66%) regardless of the tumor histological type [15]. This model showed a better false negative rate (0%) and positive rate (57.2%) than the Mayo criteria [15]. Meydanli et al. brought a "Lymph Node Metastasis Risk Index", a formula of (tumor grade) × (primary tumor diameter) × (percentage of myometrial invasion) × (preoperative serum CA 125 level), and reported it was an independent risk predictor of LNM in EC [16]. Other studies included histological type, histological grade, depth of myometrial invasion, lymph vascular space invasion (LVSI), cervical involvement, parametrial involvement, and hemoglobin levels to predict LNM of EC [17–19]. However, whether these indicators comprehensively assess LNM risk has not been determined.

Epidemiological studies have reported that the risk of EC is associated with a single factor in metabolic syndrome (MetS), including obesity, type 2 diabetes, and hypertension [20]. Diabetes mellitus showed a significant association with the presence of cancer coexistent with endometrial hyperplasia (OR = 1.96; 95% CI, 1.07–3.60; *p* = 0.03), indicating that endometrial hyperplasia in patients with diabetes mellitus can hide a certain risk of containing an occult endometrial carcinoma [21]. Other cohort studies and meta-analyses support a relationship between diabetes and an increased risk of endometrial cancer [22, 23]. Cust AE et al. performed a case–control nested study within the European Prospective Investigation into Cancer and Nutrition (EPIC) and reported that the presence of MetS was associated with EC risk (RR:2.12, 95% CI:1.51–2.97) [24]. Fundamental research showed metabolic syndrome is associated with the dysfunction of lymph nodes [25]. Adipocytes and fatty acids support the survival of metastatic cancer cells, leading to cancer progression and metastatic growth [26]. A clinical study of the association between metabolic components and EC has been documented. Kho PF et al. performed a bidirectional, two-sample Mendelian randomization analysis in Europe. They assessed three major blood lipids: low-density lipoprotein (LDL) and high-density lipoprotein (HDL) cholesterol, and triglycerides of 188,577 individuals, and concluded the role of LDL and HDL cholesterol in the development of non-endometrioid EC [27]. Other studies revealed serum triglyceride levels were positively associated with the risk of EC [28]. So far, there hasn’t reported relation between metabolic indicators such as plasma lipoproteins, glucose, blood pressure, and lymphatic metastasis of EC. In some studies, indicators such as body mass index (BMI) were combined with serum CA-125 level, and MRI imaging to build a model predicting LNM, and the results varied. Wissing M et al. investigated the relationship between BMI and lymphatic metastasis in obese EC patients and reported that pelvic lymph node involvement was negatively correlated with BMI [29]. In a word, the role of metabolic indicators in LNM hasn’t been fully investigated, and establishing a reliable prediction model integrating metabolism and crucial variables may help LNM risk stratification. This study aimed to explore the predictive role of metabolic indicators in lymph node metastasis of endometrial cancer and build a predictive model.

## Methods

### Patients and study design

We conducted a cross-sectional study according to the Strengthening the Reporting of Observational Studies in Epidemiology (STROBE) reporting guideline (available at www.strobe-statement.org). In our study, a total of 1076 patients who underwent comprehensive surgical staging with pelvic lymphadenectomy between January 2004 and December 2020 were included. The inclusion criteria were as follows: (1) underwent surgical staging and postoperative histologically diagnosed with endometrial cancer (2) did not receive other treatments such as radiotherapy, chemotherapy, and hormones before surgery (3) informed consent was obtained. The exclusion criteria were: (1) accompanied by secondary malignancies (2) patients with other severe diseases (3) young patients who chose fertility preservation (4) incomplete clinical information. NCCN guidance of EC recommends surgical staging (hysterectomy and bilateral salpingo-oophorectomy with pelvic and paraaortic lymphadenectomy) for medically operable cases [12]. Preoperative clinical staging indicators such as myometrium invasion and distant metastasis were evaluated by MRI and CT. We adopted appropriate inclusion and exclusion criteria to guarantee the selection of representative samples and the extrapolation of the results. To assess metabolic indicators’ predictive potential in LNM, all cases who underwent staging surgery were included in our cohort. To avoid selection bias such as Neyman bias, all patients including death cases meeting inclusion criteria were included and patients were then randomly divided into two groups in a 2:1 ratio, to form a training cohort (*N* = 717) and a validation cohort (*N* = 359). A preoperative blood test of 1076 patients was collected, including biochemical values of serum fasting blood glucose (FBG), Cholesterol, triglyceride (TG), high-density lipoprotein (HDL), and diabetes mellitus (DM). Clinical information was collected, including age, BMI, systolic blood pressure (SBP), diastolic blood pressure (DBP), pulse pressure (PP), hypertension (HP), and menopause status. Post-operation pathological indicators were collected, such as tumor grade, myometrium invasion (MI), cervical invasion (CI), LNM, tumor size, LVSI, peri-cytology, and histological type. Metabolic syndrome risk (MSR) was constructed using serum metabolite level, age, and BMI to comprehensively evaluate metabolic risk [30]. Univariate and multivariate logistic regression was performed on the above indicators.

### Statistical analyses

Clinical information includes age, BMI, SBP, DBP, PP, FBG, Cholesterol, TG, HDL, MSR, DM, HP, menopause status, peri-cytology, LVSI, histological type, grade, MI, CI, LNM, tumor size. Univariate and multivariate logistic regression analyses were used to identify independent risk factors predictive of LNM. The significant factors in multivariant logistic regression were included to develop the nomogram. This nomogram includes line segments representing variables, graphically demonstrating muti-cox regression analysis results. The total score was obtained by adding each variable point. Then, the probability of LNM can be located on the line chart. This nomogram transforms the regression analyses into a visual chart, making the results of the prediction model more convenient for clinical practice. The performance of the nomogram was assessed in both the training and validation groups. The receiver operating characteristic (ROC) curve of the nomogram and Mayo criteria was calculated. The ROC curve reflects the accuracy and specificity of the model by calculating the area under the curve. The larger the area under the curve, the higher the accuracy and specificity of the model. A calibration plot was conducted to show the accordance between the prediction model and actual outcomes. Decision curve analysis (DCA) was performed to measure the clinical utility of the nomogram [31]. Net benefit analysis measures the benefits and harms brought by a decision. The horizontal axis is the set probability threshold, exceeding which the LNM may occur. The vertical axis is the net benefit (NB) after subtracting the harms. The model with the highest net benefit at a particular threshold probability has a higher clinical value and may bring better clinical consequences [32]. The analyses were performed by SPSS 21.0 and R software version 3.4.4 (https://www.r-project.org/), using the “rms, presence/absence, and decision curve” packages. *P* < 0.05 was considered statistically significant.

## Results

### Clinical characteristics of patients

**Fig. 1:**
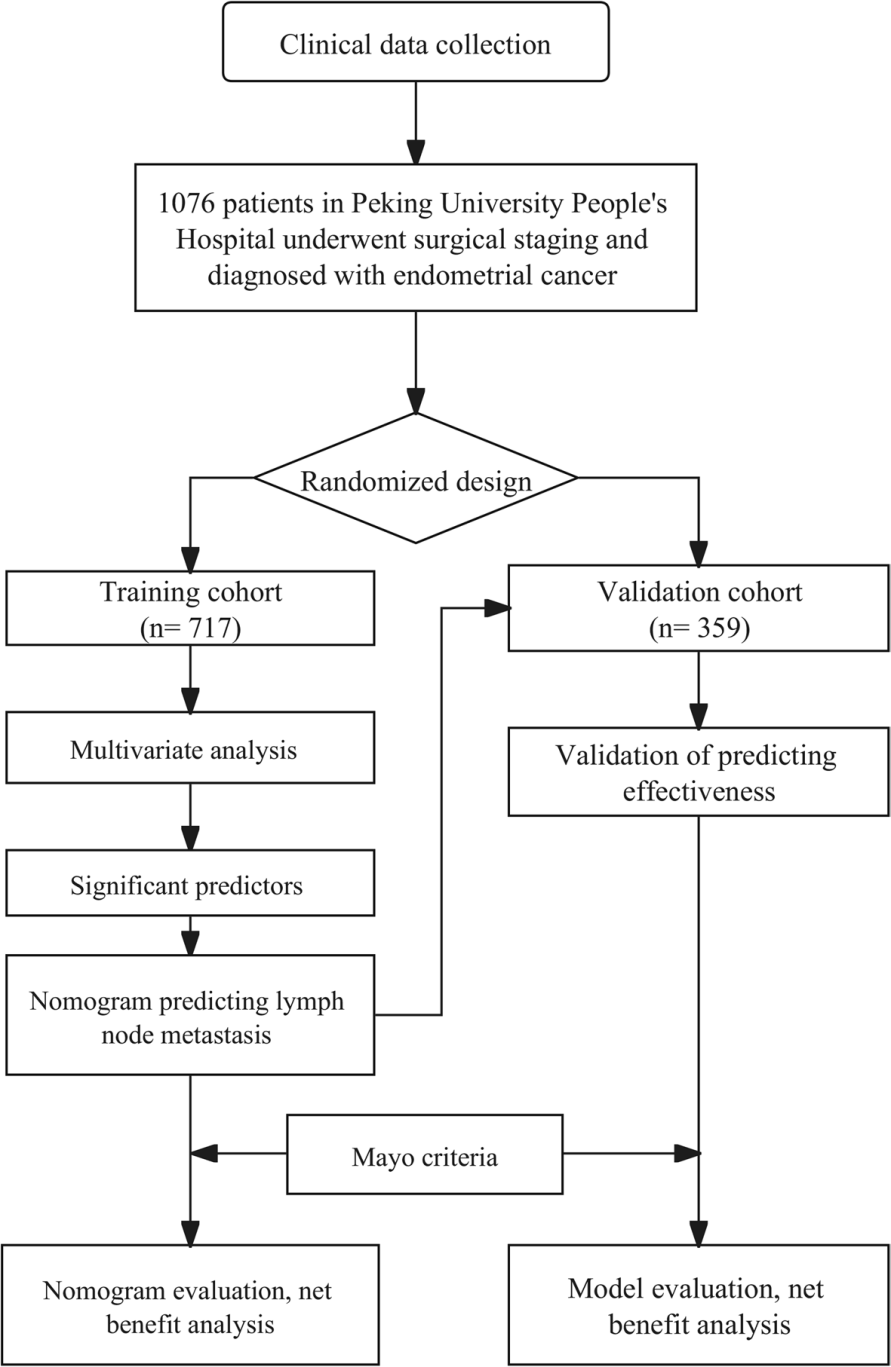
Flowchart of study design. 1076 patients were divided into training and validation cohorts according to the ratio of 2:1. Uni-cox and multi-cox regression analyses were conducted to screen significant indicators

The data from a total of 1076 patients were included in the study. Out of the patients, 717 patients were placed within the training cohort, while 359 were placed within a validation cohort. The cohort and analysis process was summarized in the flowchart (Fig. 1). The mean ages of patients within the training and validation sets were 56.27 ± 9.53 and 55.87 ± 9.26 years, respectively. Blood pressure and serum metabolite indicators, including cholesterol, HDL, and TG, were collected. The mean of BMI, SBP, DBP, PP, FBG, cholesterol, TG, and HDL of the two cohorts was summarized in Table 1. MSR was calculated based on criteria (Table S1 in the Supplement). We also included diabetes mellitus (21.06% of training cohort, 23.12% of validation cohort), and hypertension (41.7% of training cohort, 39.55% of validation cohort) cases in the study. Over 60% of the cases were post-menopause in two groups. The histological type was mainly EEA. Most cases showed pathological characteristics including negative peri-cytology (93.61% of training cohort, 94.32% of validation cohort), negative LVSI (83.54% of training group, 82.45% of validation group), < 50% myometrium invasion (77.27% of training group, 76.04% of validation group). Very few cases showed lymph node metastasis (7.53% of the training group, 5.68% of the validation group). The two sets showed similar results for nearly all variables. The baseline and clinicopathologic characteristics were summarized in Table 1.

**Table 1:** The baseline and clinicopathologic characteristics

	Training cohort (*n* = 717)	Validation cohort (*n* = 359)
	mean ± SD	mean ± SD
Age (years)	56.27 ± 9.53	55.87 ± 9.26
BMI	26.30 ± 4.42	26.09 ± 4.66
SBP (mmHg)	128.17 ± 16.06	128.25 ± 15.96
DBP (mmHg)	78.84 ± 9.79	78.53 ± 9.41
PP (mmHg)	49.33 ± 13.00	49.72 ± 13.13
FBG (mmol/L)	5.95 ± 1.77	5.95 ± 1.50
Cholesterol (mmol/L)	4.96 ± 1.11	4.99 ± 1.02
TG (mmol/L)	1.57 ± 0.93	1.68 ± 0.86
HDL (mmol/L)	1.24 ± 0.32	1.23 ± 0.30
MSR	2.36 ± 4.25	2.59 ± 4.42
	N (%)	N (%)
DM		
No	566 (78.94%)	276 (76.88%)
Yes	151 (21.06%)	83 (23.12%)
HP		
No	418 (58.30%)	217 (60.45%)
Yes	299 (41.70%)	142 (39.55%)
Menopause status		
No	253 (35.29%)	118 (32.87%)
Yes	464 (64.71%)	241 (67.13%)
Peri-cytology		
Negative	659 (93.61%)	332 (94.32%)
Positive	45 (6.39%)	20 (5.68%)
LVSI		
Negative	599 (83.54%)	296 (82.45%)
Positive	118 (16.46%)	63 (17.55%)
Histological type		
EEA	654 (91.21%)	332 (92.48%)
Others	63 (8.79%)	27 (7.52%)
Grade		
1	262 (36.54%)	136 (37.88%)
2	316 (44.07%)	160 (44.57%)
3	139 (19.39%)	63 (17.55%)
MI		
< 50%	554 (77.27%)	273 (76.04%)
> = 50%	163 (22.73%)	86 (23.96%)
CI		
Negative	640 (89.26%)	324 (90.25%)
Positive	77 (10.74%)	35 (9.75%)
LNM		
Negative	663 (92.47%)	332 (94.32%)
Positive	54 (7.53%)	20 (5.68%)
Tumor Size (cm)		
< 2	295 (42.57%)	154 (44.90%)
> = 2	398 (57.43%)	189 (55.10%)

*BMI* Body mass index, *SBP* Systolic blood pressure, *DBP* Dilation blood pressure, *PP* Pulse pressure, *FBG* Fasting blood glucose, *TG* Triglycerides, *HDL* High-density lipoprotein, *LDL* Low-density lipoprotein, *TNM* Tumor node metastasis, *MSR* Metabolic risk score, *DM* Diabetes mellitus, *HP* Hypertension, *LVSI* Lymph-vascular space invasion, *EEA* Endometrioid endometrial adenocarcinoma, *MI* Myometrial invasion, *CI* Cervical stromal invasion, *LNM* Lymph node metastasis

### Risk factors for lymph node metastasis

Our univariate analyses considered age, BMI, DM, HP, menopause status, FBG, cholesterol, TG, HDL, MSR, DM, HP, Menopause status, peri-cytology, LVSI, pathology histological type, grade, MI, CI, LNM, tumor size > 2 cm as potential risk factors for LNM from the training cohort data. After multivariate logistic regression analysis, it was found that independent risk factors associated with LNM include MSR, positive peri-cytology, positive LVSI, histological type, grade, positive MI > 50%, positive cervical stromal invasion, tumor size > 2 cm, and tumor grade (Table 2). In training groups. Among these independent risk factors, LVSI was considered a major predictor (OR = 6.69, 95% CI:3.42–13.05, *P* = 0.00). Other factors considered to be predictors of LNM included MSR (OR = 1.081, 95% CI:1.01–1.16, *P* = 0.03), tumor grade 3 (OR = 1.78, 95% CI: 1.10–2.87, *P* = 0.02), positive myometrial invasion (OR = 1.86, 95% CI:1.17–2.98, *P* = 0.01). Univariate analysis and multivariate logistic regression of the training group were shown in Table 3.

**Table 2:** Univariate and multivariate analysis of lymph node metastasis in the training cohort

	Univariate analysis		Multivariate analysis	
	OR (95% CI)	*p* value	OR (95% CI)	*p* value
Age (years)	1.03 (1.00, 1.06)	0.06		
BMI	0.99 (0.93, 1.05)	0.73		
DM				
No	1.0			
Yes	1.21 (0.63, 2.31)	0.57		
HP				
No	1.0			
Yes	1.33 (0.76, 2.31)	0.32		
Menopause status				
No	1.0			
Yes	2.00 (1.03, 3.87)	**0.04**		
FBG (mmol/L)	1.12 (0.99, 1.27)	0.07		
Cholesterol (mmol/L)	0.88 (0.69, 1.13)	0.33		
TG (mmol/L)	1.11 (0.87, 1.41)	0.42		
HDL (mmol/L)	0.13 (0.05, 0.35)	** < 0.01**		
MSR	1.13 (1.06, 1.20)	** < 0.01**	1.08(1.010, 1.16)	**0.03**
Peri-cytology				
Negative	1.0			
Positive	3.41 (1.55, 7.52)	** < 0.01**	0.94 (0.35, 2.50)	0.90
LVSI				
Negative	1.0		1.0	
Positive	11.72 (6.44, 21.31)	** < 0.01**	6.68(3.42, 13.05)	** < 0.01**
Histological type				
EEA	1.0			
Others	3.89 (1.96, 7.73)	** < 0.01**	1.05 (0.45, 2.43)	0.92
Grade				
1	1.0		1.0	
2	2.73 (1.15, 6.49)	**0.02**	2.54 (1.06, 6.10)	0.04
3	7.99 (3.36, 19.01)	** < 0.01**	6.65 (2.67, 16.58)	** < 0.01**
MI				
< 50%	1.0			
> = 50%	3.87 (2.20, 6.82)	** < 0.01**	1.86 (1.17, 2.98)	**0.01**
CI				
Negative	1.0			
Positive	4.62 (2.45, 8.69)	** < 0.01**	1.733 (0.80, 3.76)	0.16
Tumor size (cm)				
< 2	1.0			
> = 2	4.05 (1.95, 8.43)	** < 0.01**	1.716 (0.75, 3.93)	0.20

*BMI* Body mass index, *SBP* Systolic blood pressure, *DBP* Dilation blood pressure, *PP* Pulse pressure, *FBG* Fasting blood glucose, *TG* Triglycerides, *HDL* High-density lipoprotein, *LDL* Low-density lipoprotein, *TNM* Tumor node metastasis, *MSR* Metabolic risk score, *DM* Diabetes mellitus, *HP* Hypertension, *LVSI* Lymph-vascular space invasion, *EEA* Endometrioid endometrial adenocarcinoma, *MI* Myometrial invasion, *CI* Cervical stromal invasion, *LNM* Lymph node metastasis

**Table 3:** Univariate and multivariate analysis of lymph node metastasis in the validation cohort

	Univariate analysis		Multivariate analysis	
	OR (95% CI)	*p* value	OR (95% CI)	*p* value
Age	1.03 (0.99, 1.07)	0.16		
BMI	1.00 (0.93, 1.07)	0.93		
DM				
No	1.0			
Yes	0.91(0.40, 2.07)	0.82		
HP				
No	1.0			
Yes	1.51 (0.76, 2.99)	0.24		
Menopause status				
No	1.00			
Yes	1.59 (0.73, 3.49)	0.25		
SBP (mmHg)	1.01 (0.99, 1.03)	0.19		
DBP (mmHg)	1.01 (0.97, 1.04)	0.76		
PP (mmHg)	1.02 (0.99, 1.04)	0.17		
FBG (mmol/L)	1.00(0.80, 1.25)	0.99		
Cholesterol (mmol/L)	1.12 (0.80, 1.55)	0.52		
TG (mmol/L)	1.18 (0.83, 1.69)	0.36		
HDL (mmol/L)	0.26 (0.07, 1.03)	0.06		
MSR	1.24 (1.05, 1.21)	** < 0.01**	1.11 (1.02, 1.20)	0.01
Peri-cytology				
Negative	1.00			
Positive	6.97 (2.63, 18.42)	** < 0.01**	2.04 (0.49, 8.43)	0.33
LVSI				
Negative	1.00			
Positive	11.58 (5.51, 24.33)	** < 0.01**	5.33 (2.29, 12.42)	** < 0.01**
Histological type				
EEA	1.00			
others	6.64 (2.77, 15.93)	** < 0.01**	3.26 (0.99, 10.70)	0.05
Grade				
1	1.00		1.0	
2	4.18 (1.54, 11.35)	** < 0.01**	4.34 (1.54, 12.28)	** < 0.01**
3	4.94 (1.61, 15.15)	** < 0.01**	5.01 (1.50, 16.71)	** < 0.01**
MI				
< 50%	1.00			
> = 50%	7.74 (3.73, 16.06)	** < 0.01**	3.50 (1.47, 8.27)	** < 0.01**
CI				
Negative	1.00			
Positive	8.72 (3.93, 19.38)	** < 0.01**	1.28 (0.70, 2.35)	0.43
Tumor size (cm)				
< 2	1.00			
> = 2	7.08 (2.43, 20.56)	** < 0.01**	2.51 (0.68, 9.27)	0.17

*BMI* Body mass index, *SBP* Systolic blood pressure, *DBP* Dilation blood pressure, *PP* Pulse pressure, *FBG* Fasting blood glucose, *TG* Triglycerides, *HDL*, High-density lipoprotein, *LDL* Low-density lipoprotein, *TNM* Tumor node metastasis, *MSR* Metabolic risk score, *DM* Diabetes mellitus, *HP* Hypertension, *LVSI* Lymph-vascular space invasion, *EEA* Endometrioid endometrial adenocarcinoma, *MI* Myometrial invasion, *CI* Cervical stromal invasion, *LNM* Lymph node metastasis

### Design and validation of the nomogram

**Fig. 2:**
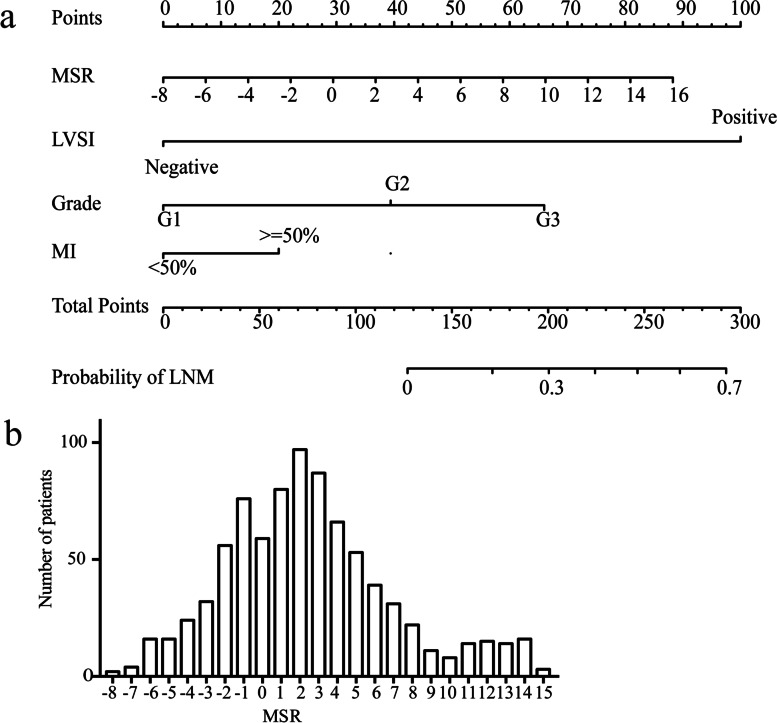
Nomogram of the model and MSR distribution. Endometrial cancer LNM prediction nomogram is depicted (**a**). For an individual, the values of each variable can be located on the segment representing indicators. A line was drawn upward to determine the point. The sum of the points responds to the likelihood of the LNM. MSR were in normal distribution among patients (**b**)

**Fig. 3:**
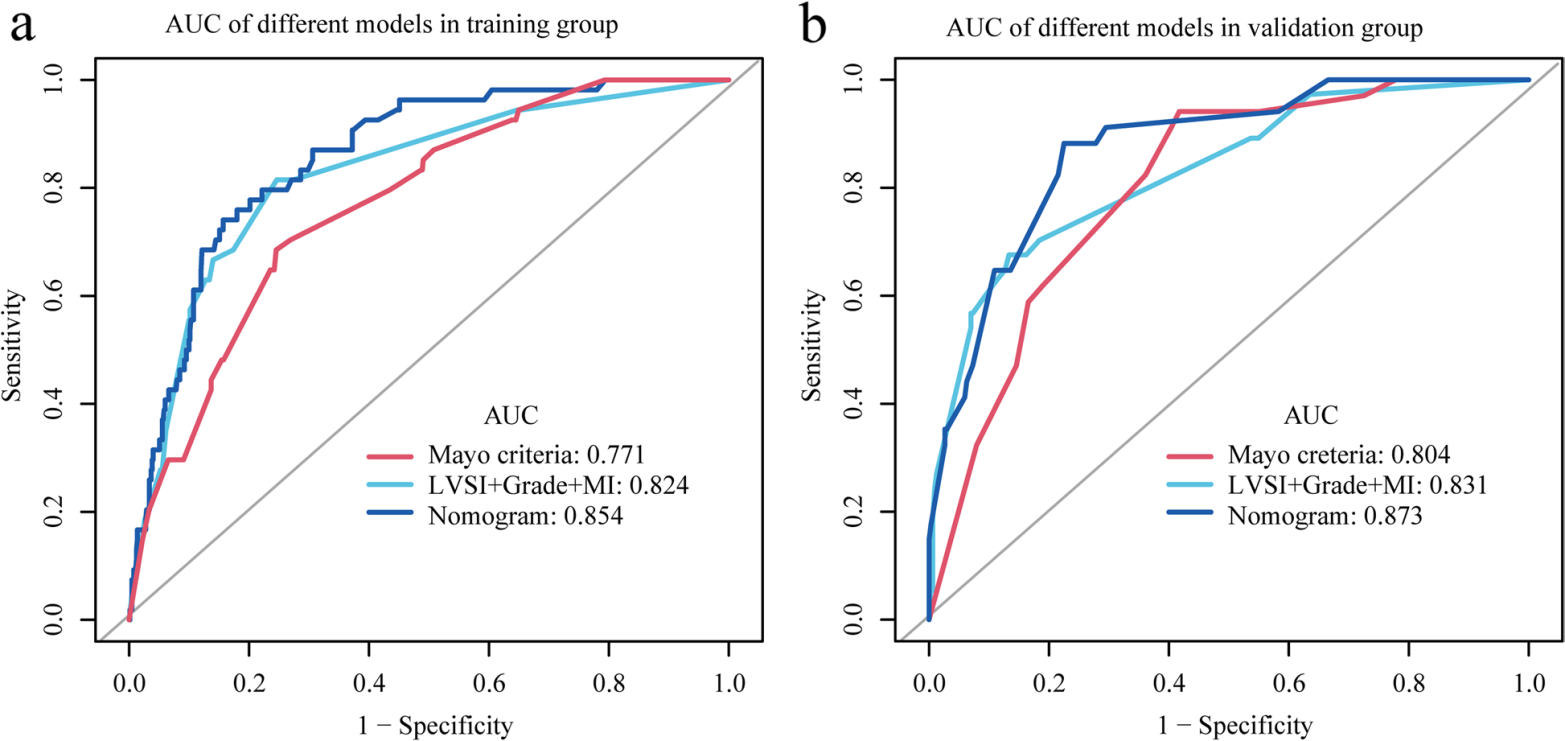
ROC analysis. AUC of Mayo criteria, a model containing three indicators, and the model we constructed in training cohort (**a**) and validation cohort (**b**)

**Fig. 4:**
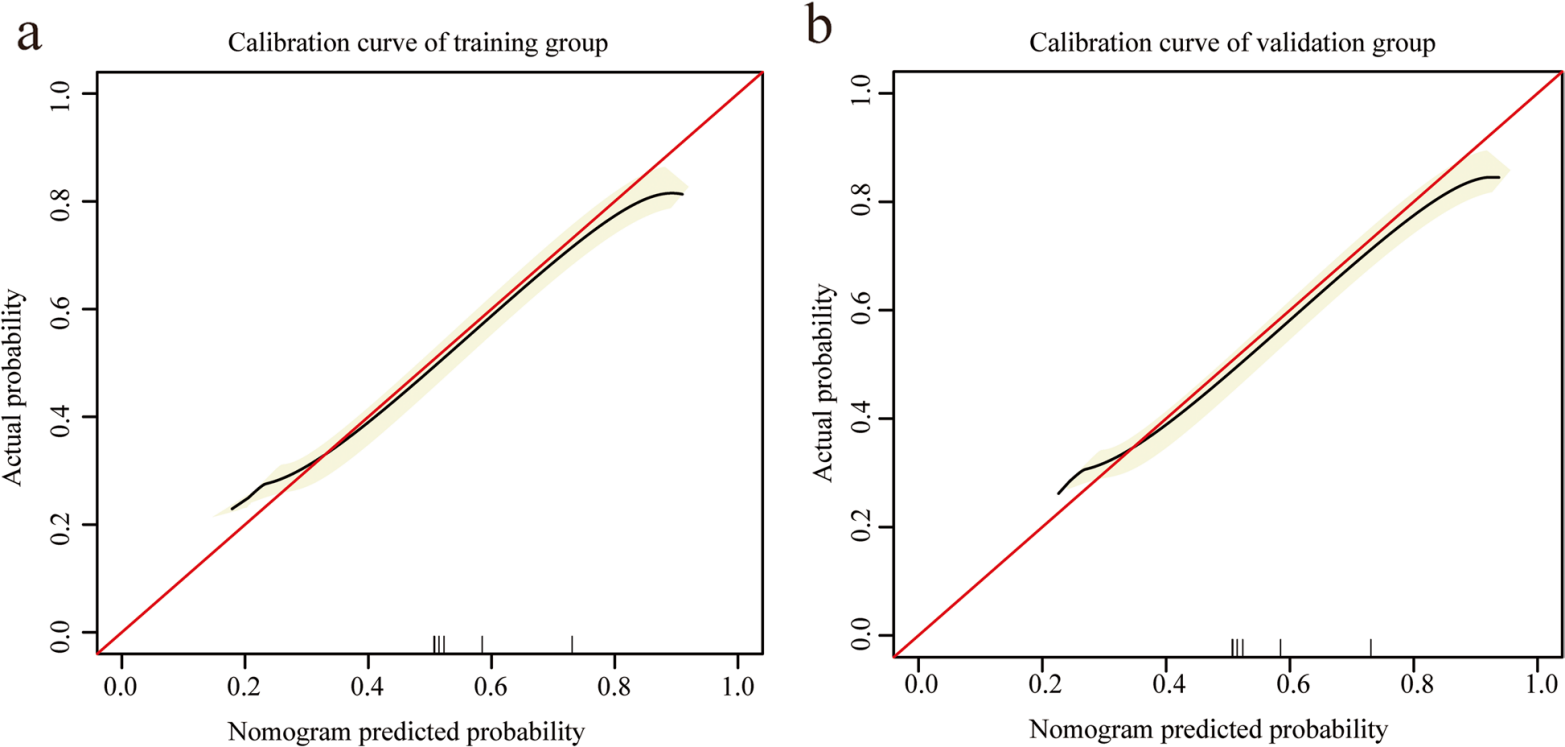
Calibration analyses of the nomogram. Calibration curves predict the overall survival of patients in the training cohort (**a**) and the validation cohort (**b**). The x-axis indicates the predicted survival probability, and the y-axis indicates the actual survival probability. The 45-degree line (gray line) indicates that the prediction agrees with actuality

Based on the independent risk factors identified in the multivariate regression analysis, we designed a nomogram to predict LNM in EC patients (Fig. 2). Among the variables considered in the predictive model, LVSI was identified to be the most important predictive factor for the LNM nomogram. Grade also showed a high-risk predictor for LNM. A scale is marked on the line segment corresponding to each variable, representing the range of the variable, and the length of the line segment reflects the contribution of the factor to the outcome. The accumulated score for each variable state represents the probability of LNM. Discrimination and calibration analyses were applied to assess the performance of the final model. The nomogram had an AUC value of 0.85 (95% CI: 0.81–0.90) for the training group, as compared with 0.77 (95% CI: 0.71–0.83) for the Mayo criteria (P < 0.01; Fig. 3a). In the validation group, the AUC value was 0.87 (95% CI: 0.82–0.93) for the nomogram and 0.80 (95% CI: 0.74–0.87) for the Mayo criteria, respectively (*P* = 0.01; Fig. 3b). The calibration curves demonstrated satisfactory probability consistencies between the prediction and observation of LNM in both the training (Fig. 4a) and validation groups (Fig. 4b).

**Fig. 5:**
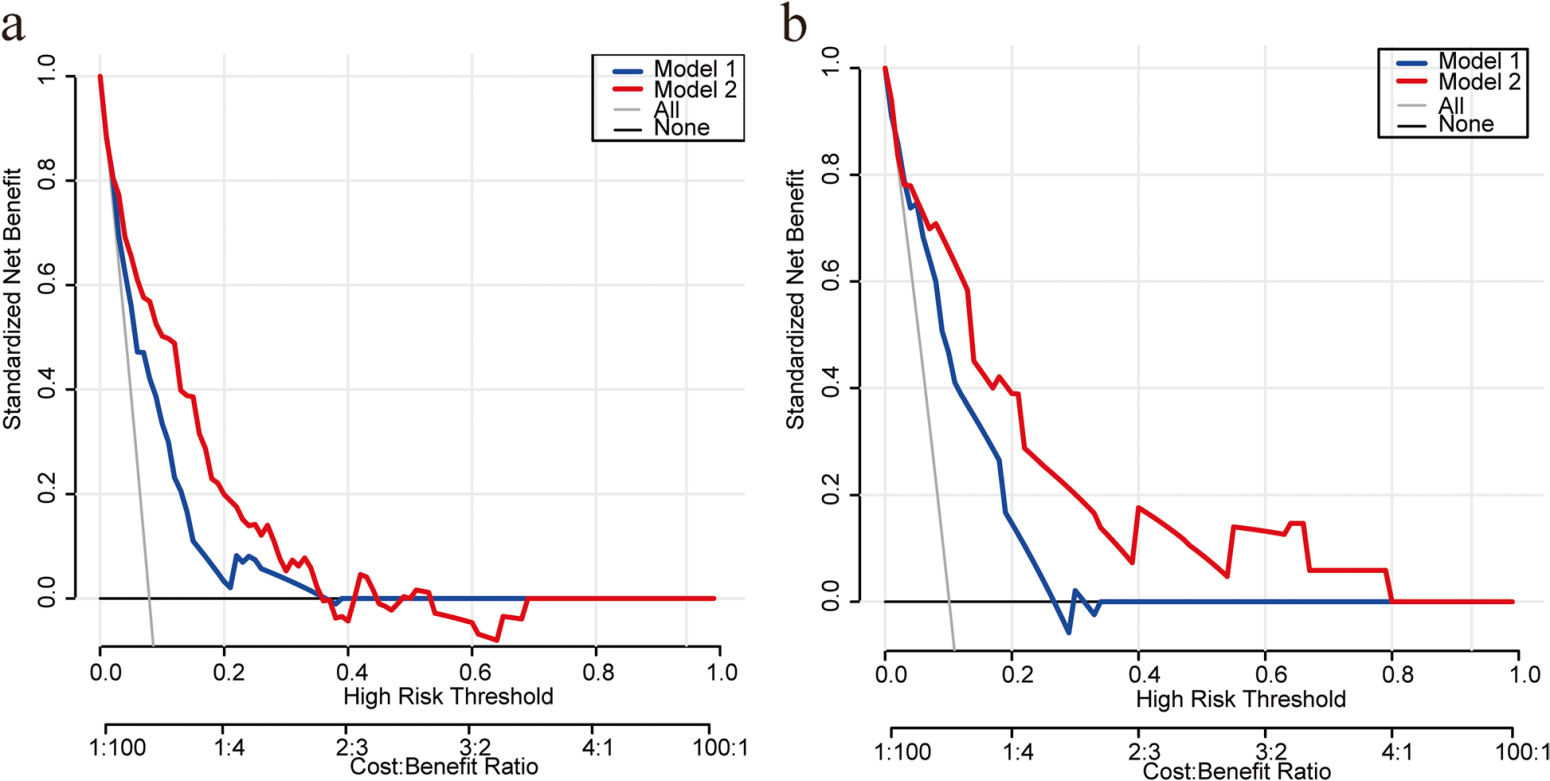
Decision curve analyses. Net benefit of Mayo criteria and the model in training cohort (**a**) and validation cohort (**b**). Model 1, Mayo criteria. Model 2, the nomogram

The performance of the nomogram was compared to the Mayo criteria for predicting LNM. In the training group, the positive predictive value was 28.17% for the nomogram and 19.37% for Mayo criteria (*P* < 0.01; Table 4). In the validation group, the positive predictive value was 30.61% for nomogram and 20.25% for Mayo criteria (*P* = 0.01; Table 5). The decision curve analysis results for the nomogram and Mayo models are shown in Fig. 5a (training cohort) and Fig. 5b (validation cohort). For predicted probability thresholds between 0% and nearly 70%, the nomogram showed a positive net benefit for the training cohort, while in the validation cohort, the threshold was 80%.

**Table 4:** The performance of the nomogram and Mayo criteria scoring system in predicting lymph node metastasis in the training cohort

Test	Mayo	Nomogram	*p* value (compare)
ROC area (AUC)	0.77	0.85	** < 0.01**
95%CI lower	0.71	0.81	
95%CI upper	0.83	0.90	
Best threshold	-2.56	-2.41	
Specificity	0.75	0.84	
Sensitivity	0.69	0.74	
Accuracy	0.75	0.83	
Positive-LR	2.79	4.56	
Negative-LR	0.42	0.31	
Diagnose-OR	6.70	14.73	
N-for-diagnose	2.27	1.73	
Positive-PV	0.19	0.28	
Negative-PV	0.97	0.97	

*AUC* The area under the receiver operating characteristic curve, *LR* Likelihood ratio, *PV* Predictive value

**Table 5:** The performance of the nomogram and Mayo criteria scoring system in predicting lymph node metastasis in the validation cohort

Test	Mayo	Nomogram	*p value* (compare)
ROC area (AUC)	0.80	0.87	0.01
95%CI lower	0.74	0.82	
95%CI upper	0.87	0.93	
Best threshold	-2.89	-2.20	
Specificity	0.58	0.78	
Sensitivity	0.94	0.88	
Accuracy	0.62	0.79	
Positive-LR	2.26	3.92	
Negative-LR	0.10	0.15	
Diagnose-OR	22.35	25.81	
N-for-diagnose	1.91	1.52	
Positive-PV	0.20	0.31	
Negative-PV	0.99	0.98	

*AUC* The area under the receiver operating characteristic curve, *LR* Likelihood ratio, *PV* Predictive value

## Discussion

Since the publication of Gynecologic Oncology Group (GOG) study 33, the risk of lymph node metastasis (LNM) in endometrial cancer has been recognized to be influenced by surgical staging, tumor grade, and depth of myometrial invasion [33]. Mayo Clinic predicts lymph node metastasis based on tumor differentiation degree, depth of myometrium invasion, tumor diameter, and histological type to guide whether to perform lymph node dissection. In 2009 ASTEC study group conducted a cohort study of 1408 EC patients from 85 centers and reported no benefit in terms of overall or recurrence-free survival for pelvic lymphadenectomy in women with early endometrial cancer [34]. A randomized clinical trial of 514 patients reported similar results, systematic pelvic lymphadenectomy did not improve disease-free or overall survival [35]. The European Society for Medical Oncology, European Society for Radiotherapy & Oncology, and The European Society of Gynecological Oncology (ESMO-ESGO-ESTRO) recommend a full staging procedure for high-risk patients (poorly differentiated with a depth of myometrium invasion > 1/2), while low-risk patients (highly differentiated and with a depth of myometrium invasion < 1/2) may not undergo lymphadenectomy [36]. As for patients in the medium-risk group, there is insufficient data, and lymphadenectomy is still recommended [36]. Overtreatment may occur in this group. Evidence suggests that women who undergo lymphadenectomy are more likely to experience surgery-related systemic morbidity or lymphoedema, which highlights the importance of carefully considering the risks and benefits of this procedure [37–39].

In recent years, SLNB has shown potential in clinical application as it reduces surgical trauma and excessive lymph node excision [40]. For low-risk patients, the consensus is that SLNB can significantly reduce the incidence of surgical complications and guide intraoperative decision-making instead of systematic lymphadenectomy [41]. For high-risk patients, SLNB had acceptable diagnostic accuracy and can guide adjuvant chemotherapy and radiotherapy [42, 43]. It is suggested that SLNB combined with ProMisE classification in the high-risk group needs more data to support [44]. Furthermore, attention should be paid to preoperative evaluation for suspicious positive lymph nodes or extrauterine invasion and metastasis as complementary to SLNB in high risk group [44].

This model aimed to predict or evaluate the risk of LNM. From the results, the nomogram has the highest AUC among three models, suggesting that comprehensive assessment is preferred, and Mayo criteria containing three indicators are not precise enough to distinguish high-risk group. The 2020 ESTRO/ESGO/ESP guidelines proposed a novel risk stratification model combining TCGA molecular signature and classic clinicopathologic prognostic factors such as MI, histological type, and LVSI to assess the prognosis of EC [45–48]. A systematic review of 6 studies with 3331 patients and a meta-analysis of 2276 patients showed LVSI has a prognostic value independent of TCGA signature (HR = 1.818, CI 95%, 1.378–2.399) [48]. The researcher explored TCGA cases and developed a 5-gene panel associated with LNM [49]. Our results suggested that LVSI is a critical factor in predicting LNM, but combining TCGA signature remains a future investigation. In some research, the factors related to LNM include histological type, pathological grade, tumor size, positive peri-cytology, involvement of adnexal and lymphatic space, etc. [11]. Previously, we reported an LNM prediction model based on a large population that performed better than the Mayo criteria [50]. Other indicators were investigated, including a score based on demographic factors, biochemical factors, and preoperative tumor characteristics [51]. These findings based on large samples and combined weighted risk factors all show relatively good predictive accuracy. To note, EC patients often present with complications such as obesity, hypertension, and diabetes, which should be considered when developing a model.

The highlight of this study is the inclusion of metabolic factors in the prediction model of lymphatic metastasis. Previous studies have reported direct associations between MetS (metabolic syndrome) and endometrial cancer risk, with women having metabolic disorders such as obesity and diabetes being at an increased risk of developing endometrial cancer [20, 52]. A case–control study nested within the European Prospective Investigation into Cancer and Nutrition (EPIC) on 284 women with endometrial cancer found that women with MetS had a relative risk for endometrial cancer (HR = 2.12, 95% CI:1.51–2.97), and there was a positive trend in risk for patients with an increasing number of Mets components [53]. Some studies have identified BMI, body fat percentage, and fat mass as independent predictors of endometrial cancer risk [54]. Metabolic dysfunction may also affect the biological behavior of endometrial cancer, as suggested by a retrospective study of 506 endometrial cancers, which found that patients with MetS had a higher positive rate of LNM, LVSI, and deep-MI proportion [52]. Based on the importance of Mets, the study comprehensively included clinical indicators to identify significant risk factors. MSR, positive peri-cytology, tumor grade, LVSI, and MI were found to be independent factors and used to build the model. MSR was normally distributed in EC patients and was a significant indicator for LMN, implying that metabolic mechanisms may be involved in EC lymph node metastasis. When evaluating the metabolic risk of patients, we referenced a metabolic risk system that modified the system introduced by the Framingham heart study. This risk score system includes BMI, PP, FBG, TG, and HDL [30]. In the current study, MSR was a significant indicator for LMN, but the components of the scoring system were not fully investigated such as PP, FBG, TG, and HDL. Some articles suggest that HDL may be a prognostic marker of EC [24, 55], but there is no direct evidence that it is associated with metastasis.

Decision curve analysis of the validation group demonstrated that when the threshold was within the range of 0.1–0.8, the nomogram showed a better net benefit compared to the Mayo criteria. Additionally, calibration curve analysis indicated strong calibration and promising predictive efficiency of the nomogram. Compared to the Mayo criteria, this model had higher sensitivity, indicating that it could effectively stratify patients. The negative predictive value (NPV) refers to the proportion of actual negative samples over all predicted negative samples, reflecting the power of identifying negative samples and the reliability of excluding the LNM. Our results showed that Mayo criteria and our nomogram have high NPV. The specificity refers to the percentage of predicted negative samples among actual negative samples. Compare with Mayo criteria, this nomogram exhibited higher specificity and accuracy in both training and validation groups. Given that MSR is a risk factor in LNM, evaluating and intervening in metabolic status might be a promising strategy for improving the clinical outcomes of EC patients.

Artificial intelligence (AI) has shown great promise in the field of gynecologic malignancies. Several studies have demonstrated that AI algorithms can be effective in diagnosis. Yan et al. constructed an MRI radiomics model and help radiologists to improve the assessments of pelvic lymph node metastasis in EC preoperatively [56]. The deep learning network model derived from MR imaging provided a competitive, time-efficient diagnostic performance in myometrium invasion depth identification [57]. Xu et al. reported that AI algorithms exhibited favorable performance for the diagnosis of ovarian cancer through medical imaging [58]. Erdemoglu et al. also used AI to identify women at risk of endometrial intraepithelial neoplasia and endometrial cancer, they selected 3 indicators by the Boruta algorithm for use in the final modeling [59]. AI-based approaches have been applied to other gynecological diseases. The deep learning model showed potential for excluding adenomyotic uteri, with higher specificity and NPV than those of intermediate-skilled trainees [60]. Guerriero et al., tested the following models: k-nearest neighbors algorithm, Naive Bayes, Neural Networks, Support Vector Machine, Decision Tree, Random Forest, and Logistic Regression in the accuracy of ultrasound soft markers identifying rectosigmoid deep endometriosis [61]. Other researchers have studied preoperative assessment such as CA125 testing, and immune cell composition for evaluating the risk of LNM [62–64]. In light of these promising results, it is possible that AI algorithms could play a valuable role in predicting lymph node involvement in endometrial cancer and contribute to more accurate and personalized treatment decisions.

The limitation of this study is that it was single-center and retrospective. Future research with an increased sample size would provide valuable information. Despite these limitations, developing a predictive model has great significance in EC management and patient counseling. With the help of a nomogram, the low-risk group can choose to undergo more conservative treatment to avoid pelvic lymph node dissection and improve the quality of life, and high-risk patients can be identified and receive aggressive treatment plans.

## Conclusion

We developed a nomogram for predicting lymph node metastasis in endometrial cancer and evaluated the effectiveness and net benefit of the model. The establishment of this tool can facilitate personalized and precision therapy approaches for endometrial cancer, thereby improving its prognosis.

## Supplementary Information

**Additional file 1: Table S1.** Metabolic scoring system.

## Data Availability

The data that support the findings of this study are available from the corresponding author upon reasonable request.
