# Supplementary material for: Metabolic syndrome score as an indicator in a predictive nomogram for lymph node metastasis in endometrial cancer
Source: BMC Cancer. 2023 Jul 4;23:622. doi: 10.1186/s12885-023-11053-4 (PMC10318658; doi:10.1186/s12885-023-11053-4)
Supplement: Supplementary file 1 — Additional file 1: Table S1. Metabolic scoring system. [file 12885_2023_11053_MOESM1_ESM.docx]

## Supplementary Materials

### TABLE S1. Metabolic scoring system.

| **Metabolic risk factor** | **Range** | **Score** |
| --- | --- | --- |
| BMI (kg/m^2^) | Q1: 17.15-19.72 | -1 |
|  | Q2: 19.72-21.22 | 0 |
|  | Q3: 21.22-22.60 | 0 |
|  | Q4: 22.60-24.77 | 1 |
|  | Q5: 24.77-29.05 | 1 |
| PP (mmHg) | Q1: 25.00-35.00 | -2 |
|  | Q2: 35.00-40.00 | -1 |
|  | Q3: 40.00-50.00 | 0 |
|  | Q4: 50.00-55.00 | 1 |
|  | Q5: 55.00-80.00 | 2 |
| FBG (mmol/L) | Q1: 3.76-4.51 | -2 |
|  | Q2: 4.51-4.93 | -1 |
|  | Q3: 4.93-5.52 | 0 |
|  | Q4: 5.52-7.47 | 2 |
|  | Q5: 7.47-13.69 | 9 |
| TG (mmol/L) | Q1: 0.41-0.67 | 0 |
|  | Q2: 0.67-0.87 | 0 |
|  | Q3: 0.87-1.08 | 0 |
|  | Q4: 1.08-1.51 | 0 |
|  | Q5: 1.51-3.56 | -1 |
| HDLC (mmol/L) | Q1: 0.47-0.78 | 5 |
|  | Q2: 0.78-0.96 | 3 |
|  | Q3: 0.96-1.18 | 2 |
|  | Q4: 1.18-1.45 | 0 |
|  | Q5: 1.45-2.04 | -3 |
|  |  |  |

Table S1. Metabolic scoring system. The metabolic risk score of each patient was calculated based on BMI, PP, FBG, TG, and HDLC. BMI, body mass index; PP, pulse pressure; FBG, fasting blood glucose; TG, triglycerides; HDLC, high-density lipoprotein cholesterol.
